# Lignin Quantification of Papyri by TGA—Not a Good Idea

**DOI:** 10.3390/molecules26144384

**Published:** 2021-07-20

**Authors:** Florian Bausch, Dickson D. Owusu, Paul Jusner, Mario J. Rosado, Jorge Rencoret, Sabine Rosner, José C. del Río, Thomas Rosenau, Antje Potthast

**Affiliations:** 1 Department of Chemistry, Institute of Chemistry for Renewable Resources, University of Natural Resources and Life Sciences Vienna (BOKU), Konrad-Lorenz-Straße 24, A-3430 Tulln, Austria; florian.bausch@boku.ac.at (F.B.); dickson.owusu@boku.ac.at (D.D.O.); paul.jusner@boku.ac.at (P.J.); thomas.rosenau@boku.ac.at (T.R.); 2 Instituto de Recursos Naturales y Agrobiología de Sevilla, CSIC, Avenida Reina Mercedes, 10, E-41012 Seville, Spain; m.rosado@irnas.csic.es (M.J.R.); jrencoret@irnase.csic.es (J.R.); delrio@irnase.csic.es (J.C.d.R.); 3 Department of Integrative Biology and Biodiversity Research, Institute of Botany, University of Natural Resources and Life Sciences Vienna (BOKU), Gregor-Mendel Straße 33, A-1180 Vienna, Austria; sabine.rosner@boku.ac.at

**Keywords:** papyrus, lignin, quantification, TGA, DTG, Klason-lignin, ABSL, conservation

## Abstract

Papyri belong to the oldest writing grounds in history. Their conservation is of the highest importance in preserving our cultural heritage, which is best achieved based on an extensive knowledge of the materials’ constituents to choose a tailored conservation approach. Thermogravimetric Analysis (TGA) has been widely employed to quantify cellulose and lignin in papyrus sheets, yielding reported lignin contents of 25% to 40%. In this work, the TGA method conventionally used for papyrus samples was repeated and compared to other lignin determination approaches (Klason-lignin and acetyl bromide-soluble lignin). TGA can lead to a large overestimation of the lignin content of commercial papyrus sheets (~27%) compared to the other methods (~5%). A similar overestimation of the lignin content was found for the pith and rind of the native papyrus plant. We concluded that the TGA method should, therefore, not be used for lignin quantification.

## 1. Introduction

In the context of the preservation of valuable historical objects from plant sources, such as paper, garments, furniture, or tapestry, a proper understanding of the ratio of the main constituents—cellulose, hemicelluloses, and lignin—is crucial to assess the status of conservation and to recommend suitable conservation treatments. A particularly illustrative example demonstrating this necessity is the Viking Ships in the Oseberg collection: a treatment with alum about 100 years ago led to an almost complete acidic degradation of the polysaccharide constituents, leaving the conservators no other choice than to try to preserve at least the remnants of conservation agents and lignin [1]. Another case is the Annals of the Joseong Dynasty, a thousand-year account of court life in Ancient Korea and the longest continuously written report in history. Attempts to stabilize the aging, brittle material with beeswax were of only short-lived benefit: mold that fed on the wax’s triglycerides decomposed the “preserved” papers much faster than the untreated ones [2].

Material analysis of ancient papyri started comparatively early, at the latest in 1821, when Humphry Davy reported a series of analyses on papyri from Herculaneum, including pyrolysis and slow combustion—a predecessor, if you will, to modern TGA, but in any case, a means to determine the ash content (5% to 6%) [3].

One of the first and potentially most influential lignin quantifications for modern and ancient papyrus sheets was published by Wiedemann and Bayer in 1983, based on thermogravimetric analysis (TGA), see Table 1. This approach has often been repeated, cited, and discussed rather uncritically [4,5,6,7]. The reported lignin content of 22% to 32% percent has ever since shaped the conception of conservators and conservation scientists about the lignin content of papyrus, see Table 1. Papers with a similar lignin content were even chosen as model substrates for an accelerated aging study, because it was assumed that this material composition resembled that of papyri [8].

The results in Table 1 present large differences with regard to lignin content, depending on the method used. While Klason Lignin and ABSL yielded values mostly around 15% lignin content, TGA-quantification results gave double the lignin content or even more. Lucejko et al. [12] outnumbered even these values for the lignin content of fresh papyrus plants (“middle of the stem”), reporting 46.9% lignin, determined by Pyrolysis–Gas Chromatography/Mass Spectrometry (Py-GC/MS). For modern papyrus sheets, they reported a lignin content of 29%. For their calculation, the peaks found in the chromatograms were assigned either to cellulose or to lignin. The contributions of hemicellulose, extractives, or inorganic matter were not taken into consideration regarding the total weight of the sample, hence, lignin (and cellulose) contents were increased compared to other methods.

Lignin quantification by TGA has been particularly appreciated by conservation scientists due to its low sample demand of below 10 mg, as opposed to up to 10 g for Klason lignin determination according to the TAPPI standard for pulp [17], which determines the lignin contents after acidic degradation of the polysaccharides. Such sample requirements are clearly not feasible for historical specimens, especially in the case of valuable objects. Another lignin determination approach, known as the ABSL (acetyl bromide soluble lignin) method [18,19], is based on derivatization and solubilization of the lignin and subsequent determination by UV/Vis spectroscopy (absorption band at 280 nm). Although this approach is similarly economical to TGA in terms of sample requirements, it has never been applied in papyrus conservation studies.

In this study, lignin quantification of papyrus samples was addressed. The TGA-quantification approach was repeated and studied in detail. The results were compared to the values obtained by the Klason lignin and ABSL methods. According to ancient sources, papyrus sheets should almost completely consist of papyrus pith [20], but more recent literature is not always clear about which parts of the papyrus stem had been used in their analytical studies [16]. Therefore, also papyrus rind was included in the study, along with papyrus pith and commercial sheets, both from Qaramos, Egypt.

**Figure 1:**
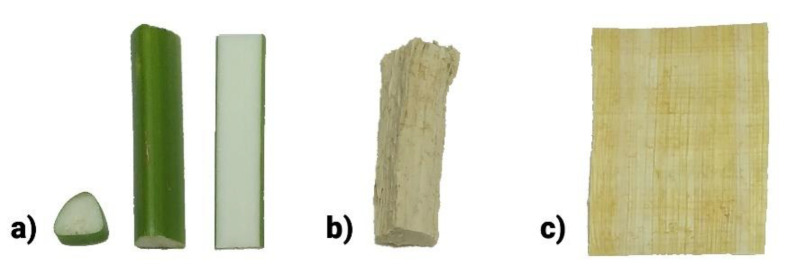
(**a**) Freshly cut papyrus stems in cross-section, part of a stem, and the stem cut in a longitudinal direction, as it is done for papyrus sheet production. (**b**) Dried papyrus pith, after the removal of the green rind. (**c**) A commercial papyrus sheet.

Figure 1 depicts the sample material used in this study. Rind was removed using a razor blade or a nylon thread. Rind and pith were dried, as shown for native pith in Figure 1b. The final product of contemporary Egyptian papyrus sheet manufacture, shown in Figure 1c, was analyzed as well, although one might expect the final sheet to have the same lignin content as native pith, since historic sources indicate no production step that would cause significant lignin removal [21].

## 2. Results and Discussion

### 2.1. Re-Evaluation of TGA—Literature Values and Comparison to Reference Materials

**Figure 2:**
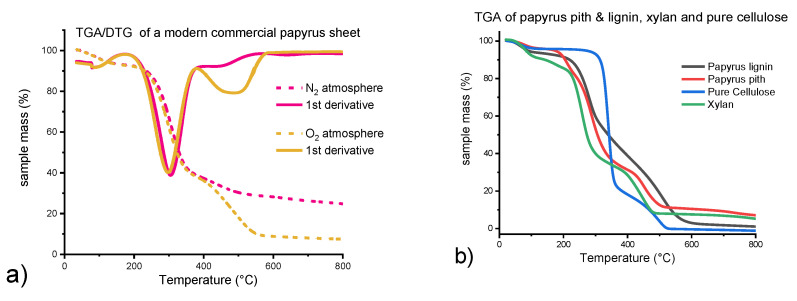

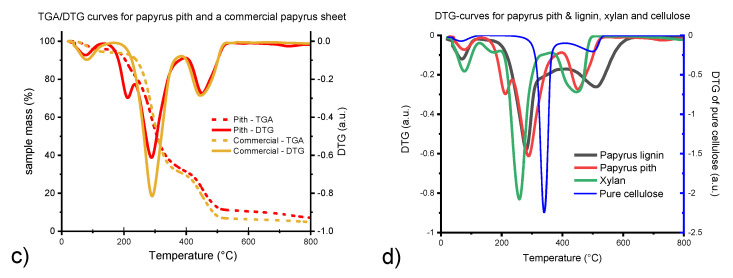
(**a**) Thermogravimetry (TGA) and derivative thermogravimetry (DTG) curves for a commercial papyrus sheet sample in nitrogen and oxygen atmosphere; (**b**) TGA curves (oxygen atmosphere) for papyrus lignin, papyrus pith, pure cellulose (Whatman filter paper No. 1), and a xylan sample. (**c**) TGA/DTG curves (oxygen atmosphere) for native papyrus pith and a commercial papyrus sheet (obtained one year later from the same supplier as the sample in (**a**)). (**d**) DTG curves (oxygen atmosphere) for papyrus lignin, papyrus pith, xylan, and pure cellulose (blue). DTG for pure cellulose is depicted on the right *Y*-axis (blue, different scale).

In order to examine how the literature values had been obtained, the TGA parameters of Wiedemann and Bayer were re-applied. Samples of papyrus sheets, pith, and rind of the papyrus plant, were heated up to 1000 °C in an oxygen atmosphere and, for comparison, in an inert nitrogen atmosphere (Figure 2a,c) at a heating rate of 10 K/min. The weight loss was recorded. To study the behavior of the individual components of papyri, the same conditions were applied to pure cellulose (Whatman filter paper No.1), hemicellulose (xylan), and papyrus lignin, extracted as “milled wood” lignin preparations (MWL) of *Cyperus papyrus* L. pith, according to [22,23] (Figure 2b,d).

The TGA curve shapes reported in the literature (Wiedemann and Bayer, 1983) for measurement in oxygen atmosphere were confirmed (Figure 2a). Thermal degradation of papyrus samples, after initial water removal above 100 °C, reached a maximum at 300 °C in a defined step, which became better observable as a distinct minimum in the first-derivative plot of the degradation curve (derivative thermogravimetry, DTG). In an oxygen atmosphere, there is a second distinct minimum around 500 °C, which is largely reduced for measurements under nitrogen (Figure 2a). In the literature cited in Table 1 [4,9,10,11], the first minimum at 300 °C was assigned to cellulose, the second minimum at about 500 °C to lignin. Quantification was done by integrating the respective peaks in the DTG plot.

As is evident from the degradation curves of the individual components (Figure 2b), there is no clear step attributable to lignin degradation. Papyrus lignin shows a degradation pattern that is to some extent similar to that of papyrus pith in the lower temperature range with a minimum around 270 °C in the DTG-plot (Figure 2d). At higher temperatures, it exhibits a very broad, gradual degradation curve, whereas cellulose degradation is characterized by a rather steep and sharp degradation step around 350 °C (Figure 2b,d). The second region of slower degradation of lignocellulosic materials, between 350 and 500 °C, gives rise to a second, broader minimum around 480 °C in the first derivative. This minimum has been wrongly assigned to lignin in previous literature (compare Table 1). It originates from further oxidation of dehydration, degradation, and condensation products of cellulose and hemicelluloses, which are usually denoted as humins. These compounds, mostly ladder-type oligomers with mixed furanoid and benzoid moieties [24], are typical products of pyrolytic and also acid-catalyzed processes of pyranoses and furanoses. They degrade more slowly in oxygen than the initial cellulose and give rise to stable graphenoid residues in the absence of oxygen. The second minimum is much larger in an oxygen atmosphere (Figure 2a), which means that a huge fraction of the mass content quantified as lignin in the literature arises from combustion processes, which are not taking place in an inert atmosphere. This additional mass loss for combustion processes as compared to pyrolysis in an inert atmosphere is found for cellulose, hemicelluloses, and lignin in a similar fashion [25]. The degradation curve for papyrus is, therefore, a superposition of the individual curves from lignin and cellulose/hemicelluloses, with the graph of the former being rather broad and the graphs of the latter two being steeper (Figure 2d). The less steep shape of the curve of papyrus pith as compared to the commercial papyrus sheet sample may indicate indirectly that the sheet has a somewhat lower lignin content (resulting in a steeper curve) than the original pith. Degradation starts at lower temperatures for papyrus pith and papyrus lignin (Figure 2b), as opposed to pure cellulose, which suggests that the first region of thermal degradation of papyrus pith is caused by the contained lignin. However, this region can never serve as basis to quantify the entire lignin content, since lignin degrades in a very broad region, e.g., for papyrus lignin between 250 and 600 °C. The same alleged “lignin peak” as in native papyrus pith is obtained by combusting xylan, also with minor contributions from cellulose (Figure 2d). This illustrates clearly the faultiness of the previous assignment and, moreover, that the approach of just assigning a small area in the TGA/DTG curves solely to lignin will lead to wrong results.

### 2.2. Comparison to Other Methods of Lignin Determination (Klason-Lignin and ABSL)

To correct the lignin contents reported by the TGA approach and to check whether they range at least in a reasonable order of magnitude, we turned to alternative methods to determine lignin contents, namely the Klason lignin method (residue after carbohydrate solubilization in 72% sulfuric acid) and the acetyl bromide method (ABSL, UV absorbance at 280 nm from lignin phenols after complete derivatizing solubilization of the sample in acetyl bromide) for more precise values. In contrast to the gravimetric methods (TGA and Klason lignin) the ABSL approach makes use of a specific property of lignin, its UV absorption, to quantify it directly.

**Figure 3:**
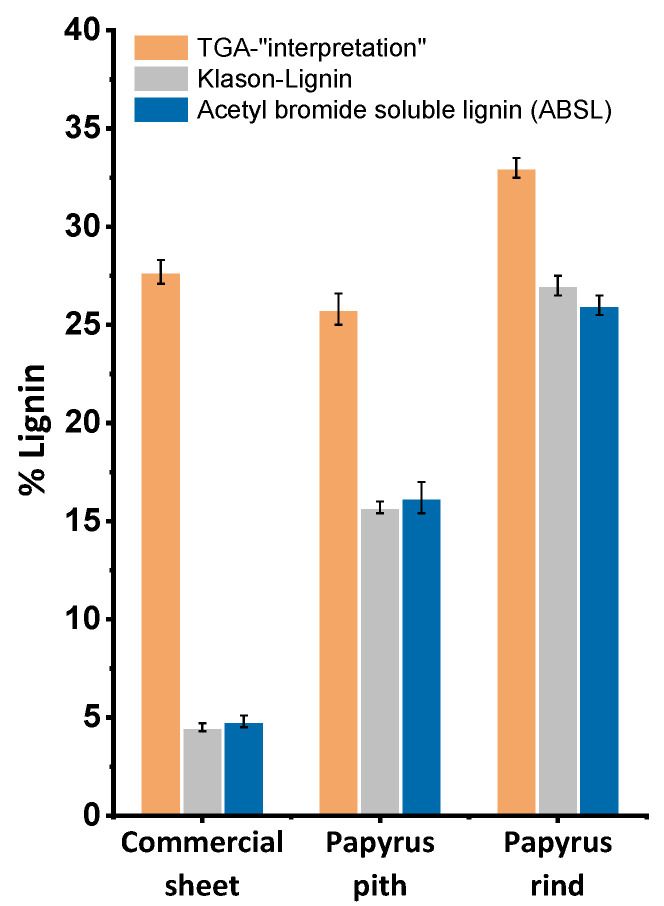
Lignin contents of a commercial papyrus sheet, native pith, and native rind, determined by TGA as compared to Klason-Lignin and acetyl bromide soluble lignin (ABSL).

As clearly shown in Figure 3, the TGA-quantification approach always led to an over-estimation regarding lignin content. This became especially drastic for the commercial papyrus sheet, where the TGA results gave a lignin content five times higher than according to the alternative methods. Klason-Lignin and ABSL provided quite consistent values throughout.

Comparing the approach of lignin quantification by TGA, as applied in the literature, to more commonly used methods of lignin quantification in the pulp and paper industry, two things become obvious. First, other substances, such as hemicelluloses, can wrongly contribute to the lignin content (Figure 2d), and second, this is not just a theoretical problem, as the lignin values of papyri reported by TGA “quantification” are far too high. Native papyrus pith contains only around 16% lignin, as opposed to 25% up to 47% following the literature. Lignin contents of commercial papyrus sheets determined according to Klason lignin and ABSL methods were, to our surprise, significantly lower than those of native pith, while rather similar contents were expected if the papyrus sheets had been produced according to ancient recipes. This huge difference was obviously not found using the TGA approach, where the curve minimum previously and wrongly used for lignin quantification shows a very similar peak area for commercial papyrus sheets and native papyrus pith (Figure 2c and Figure 3). Possible reasons for the huge decrease in lignin content when producing contemporary commercial papyri from papyrus pith will be described in a detailed upcoming account. The very low lignin content of around 5% in commercial sheets has severe implications with regard to conservation decisions, or when an appropriate material for facsimilia of ancient papyri is to be manufactured, especially if a lignin content of up to 50% is assumed based on [12].

### 2.3. General Discussion of Lignin Abundance in Papyrus Pith by Microscopic/Staining Analysis

**Figure 4:**
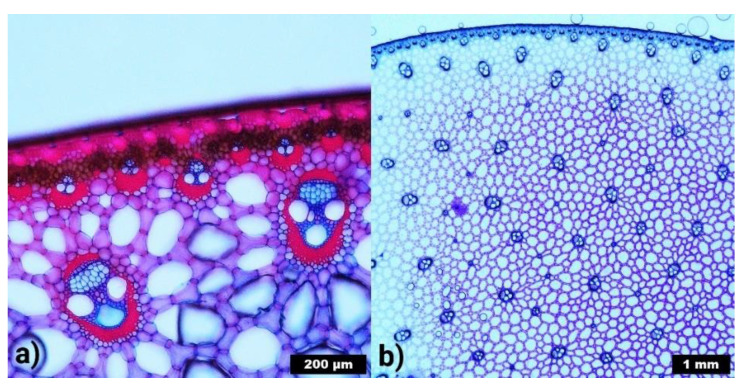
Microscopic images of cross-sections of *Cyperus papyrus* L. stems. (**a**) Detail of the outer region of papyrus pith. Lignified areas are colored red with Safranin-O, cellulosic areas are stained with Astra Blue and appear blue/pink. Scale bar 200 µm. (**b**) Overview of the structure of papyrus pith, stained with toluidine. The denser vascular bundles appear darker. Scale bar 1 mm.

Two general ideas regarding optical and structural properties of papyrus pith support the finding that lignin content in papyrus pith is much lower than in previous claims. Lignin is the “usual suspect” to color lignocellulosic materials brown, but native papyrus pith is white (compare Figure 1). The pith mainly consists of a ground tissue of parenchyma cells with big intercellular spaces, called aerenchyma, which is typical for aquatic plants (Figure 4b). Within the aerenchyma, several vascular bundles can be found. Structurally, lignin in papyrus stems is mainly found in the sclerenchymatous tissue of the vascular bundles. This lignified tissue strengthens the vascular bundles, which enable the transport of water, nutrients, and carbohydrates in the papyrus stem. The sclerenchyma can be colored bright red with the Safranin-O dye (Figure 4a). As seen in Figure 4a, a lot more lignified tissue is found in the rind, which explains the different values of lignin content between rind and pith. The rind consists of the epidermis, the sclerenchymatous hypodermis, and the cortical chlorenchyma, which results in its green color (Figure 1). The number of vascular bundles in/next to the chlorenchyma is higher but their size is smaller than that of those occurring within the aerenchyma of the pith. Thus, in the rind, lignified cell walls are found not only in the more numerous vascular bundles, but also in the sclerenchyma cells of the hypodermis.

Figure 4b provides an overview of the structure of a papyrus stem in cross-section. It becomes clear that vascular bundles, despite being a most important and integral constituent of papyrus pith, do not appear in an abundance that could suggest a lignin content of up to 50% in the pith. Values around 16% seem more reasonable, especially when considering that the vascular bundles are not solely composed of lignified sclerenchymatous tissue.

## 3. Conclusions and Outlook

A previous TGA-based approach to quantify lignin in papyrus material, originating from 1983 and frequently used and cited since then, provides wrong results. It attempted to quantify lignin from DTG curves by a peak that originated from polysaccharides, or, more precisely, from the combustion of primary pyrolysis products of polysaccharides. This led to a severe over-estimation of the lignin content. While the literature contents up to 50% were far too high, values around 5% for commercial papyrus sheets and 16% for native papyrus pith were obtained by two alternative and well-matching lignin determination methods, the Klason-lignin and the acetyl bromide soluble lignin (ABSL) methods. These methods gave reproducible and reliable lignin contents for papyrus sheets as well as the pith and rind of the papyrus plant. The ABSL method is recommended for further research as it has a sample demand similarly small to TGA, which is an important issue in the field of conservation research. The use of IR spectroscopy [26] which is completely non-destructive and proved to be helpful in fast compositional analyses of bioproducts, is currently studied for the papyrus case. An upcoming study will also address the gap in lignin content between commercial papyrus sheets and native papyrus pith, as a consequence of modern-day deviations from the traditional historic procedure of papyrus manufacture.

## 4. Materials and Methods

### 4.1. Materials

*Cyperus papyrus* L. stems were acquired directly from farmers in Qaramos, Sharquia Province, Egypt. Rind was removed by slicing the fresh papyrus stems with a nylon fiber, rind and pith were subsequently air-dried for one week. Commercial papyrus sheets were obtained from farmers in the same village.

Whatman Filter paper was obtained from Sigma-Aldrich, Steinheim, Germany. Xylan was obtained from Lenzing AG, Lenzing, Austria.

Safranin-O, Astra Blue, and Toluidine were obtained from Merck KGaA, Darmstadt, Germany.

All chemicals were purchased at the best quality available.

### 4.2. “Milled Wood” Lignin (MWL) Isolation from Papyrus Pith

To obtain papyrus lignin, air-dried *Cyperus papyrus* L. pith was milled using an Ultra Centrifugal Mill ZM 200 (Retsch, Haan, Germany). Extractives were removed by Soxhlet extraction, with acetone (12 h), methanol (16 h), and hot water (12 h) as the solvents. Lignin isolation was conducted according to the standard procedure [22]. The pre-extracted papyrus pith samples were finely milled in a PM100 planetary ball mill (Retsch, Haan, Germany) for 5 h at 400 rpm in a 500 mL agate jar and agate ball bearings (20 × 20 mm). Lignin isolation was conducted using dioxane-water (90:10, *v/v*) for 3 × 12 h. The crude MWL preparation was purified as described in detail in [23]. The yield of the isolated MWL preparation was approx. 20% of the Klason lignin content of papyrus pith.

### 4.3. Lignin Content Determination by Acetyl Bromide Derivatization and UV-Detection

Commercial papyrus sheets, and native papyrus pith and rind from Egypt were milled using an Ultra Centrifugal Mill ZM 200 (Retsch, Haan, Germany). Extractives were removed by accelerated solvent extraction (ASE) on an ASE 350 (Dionex, Sunnyvale, USA), based on a literature protocol [27]. Conditions were 60 °C, 11 MPa, 15 min static time, 36 mL per cycle. The samples were extracted by two cycles of hexane and four to six subsequent cycles of acetone/water (95:5) until the filtrate was colorless.

All samples were dried for three days in a vacuum dryer (Goldbrunn 1450, Berlin, Germany) at 40 °C. The samples (2 to 5 mg in triplicates) were dissolved in a mixture of 25% (*v/v*) acetyl bromide in glacial acetic acid (0.2 mL), heated to 50 °C, and stirred for 2 h, based on [18]. The dissolved samples were added to a mixture of aqueous NaOH (1 mL, 2 M) and hydroxylamine hydrochloride (0.175 mL, 0.5 M), and the volume was adjusted to 10 mL by glacial acetic acid. Absorbance was measured at 280 nm on a Perkin Elmer Lambda 35 UV/Vis Spectrometer (Waltham, MA, USA). An extinction coefficient of 20 L/g*cm was applied [16] to calculate the amount of acetyl bromide-soluble lignin.

### 4.4. Determination of the Lignin Content by the Klason Method

The Klason lignin content was estimated as the residue after sulfuric acid hydrolysis of 300 mg of pre-extracted material (after sequential Soxhlet extraction with acetone, 12 h, methanol, 24 h, and hot water, 12 h), according to TAPPI T222 om-88 [17] and corrected for ash and protein content as previously published [23]. The acid-soluble lignin was determined, after the insoluble lignin was filtered off, by UV-spectroscopy at 205 nm according to [17], using 110 L cm^−1^ g^−1^ as the extinction coefficient. Three replicates were measured for each determination. The values displayed in Figure 3 express the sum of Klason-lignin and acid-soluble lignin.

### 4.5. Thermogravimetric Analysis

Thermogravimetric analysis was conducted on a TG 209 F1 Thermo-Microbalance (Netzsch, Selb, Germany). The dry sample material (5 to 15 mg) was heated up to 1000 °C in aluminum oxide crucibles at a rate of 10 K/min, measurements were done in triplicate. All samples were heated in an oxidizing atmosphere ($N_{2}$:$O_{2}$/4:1) of 20 mL/min and protective flow of 8 mL/min, except for the commercial papyrus sheet in Figure 2a. For the measurements in Figure 2a, the same oxidizing conditions as for the other samples were compared to an inert atmosphere applying 20 mL/min $N_{2}$ flow and 8 mL/min $N_{2}$ protective flow. The Proteus software was used for data evaluation and manipulation.

### 4.6. Microscopic Analysis

Microscopic analysis of *Cyperus papyrus* L. stems was conducted with an Olympus DX 1000 digital microscope in incident light mode. For magnification in Figure 4a, the objective lens XLOB 20× with an objective magnification of 20× was used, for magnification in Figure 4b, the objective lens XLOB 3× with an objective magnification of 3×. Cross-sections were cut with a razor blade. The native tissue was stained with Astra Blue for cellulosic tissue and Safranin-O for lignified tissue. Toluidine was applied to enhance the contrast of the entire sample.

## Figures and Tables

**Table 1:** Comparison of lignin content of papyrus sheets (or stems) reported in the literature.

Lignin Content	Method	Reference	Ash Content
22–32%	TGA	[4]	2–14%
32–40%	TGA	[9]	Subtracted
22–26%	TGA	[10]	~20%
36–40%	unspecified	[11]	Not determined
29–47%	Py-GC/MS	[12]	Not subtracted
13%	“a TAPPI method”	[13]	Not determined
12–25%	Klason	[14]	6%
17%	Klason	[15]	3%
14% (whole stem)	ABSL	[16]	Not determined

## Data Availability

Not applicable.
